# An Enriched Environment Promotes Shelter-Seeking Behaviour and Survival of Hatchery-Produced Juvenile European Lobster (*Homarus gammarus*)

**DOI:** 10.1371/journal.pone.0159807

**Published:** 2016-08-25

**Authors:** Stian Aspaas, Ellen Sofie Grefsrud, Anders Fernö, Knut Helge Jensen, Henrik Trengereid, Ann-Lisbeth Agnalt

**Affiliations:** 1 Hammervegen 3A, 7350 Buvika, Norway; 2 Institute of Marine Research, P. O. Box 1870 Nordnes, 5817 Bergen, Norway; 3 University of Bergen, Department of Biology, P. O. Box 7803, N-5020 Bergen, Norway; 4 Myrdalhovden 48, 5131 Nyborg, Norway; Evergreen State College, UNITED STATES

## Abstract

The high loss of newly released hatchery-reared European lobster (*Homarus gammarus*) juveniles for stock enhancement is believed to be the result of maladaptive anti-predator behaviour connected to deprived stimuli in the hatchery environment. Our objective was to learn if an enriched hatchery environment enhances shelter-seeking behaviour and survival. In the “naïve” treatment, the juveniles were raised in single compartments without substrate and shelter whereas juveniles in the “exposed” treatment experienced substrate, shelter and interactions with conspecifics. Three experiments with increasing complexity were conducted. Few differences in shelter-seeking behaviour were found between treatments when one naïve or one exposed juvenile were observed alone. When observing interactions between one naïve and one exposed juvenile competing for shelter, naïve juveniles more often initiated the first aggressive encounter. The third experiment was set up to simulate a release for stock enhancement. Naïve and exposed juveniles were introduced to a semi-natural environment including substrate, a limited number of shelters and interactions with conspecifics. Shelter occupancy was recorded three times during a period of 35 days. Exposed juveniles occupied more shelters, grew larger and had higher survival compared with naïve juveniles. Our results demonstrate that experience of environmental complexity and social interactions increase shelter-seeking ability and survival in hatchery reared lobster juveniles.

## Introduction

Behaviour in the early life stages can be modified through a number of abiotic and biotic factors. To survive in the wild, animals must learn basic behavioural skills that allow them to find food, shelter and avoid predation [1]. In intensive aquaculture, the larvae and juveniles are normally deprived of the environmental complexity that is assumed to ensure an appropriate behavioural repertoire to cope with the ever-changing natural habitat. In addition, aquaculture production lines select for traits that increase economic yield, such as growth and survival, but not behaviour required for survival in the wild [2]. Efforts to increase recruitment to the fisheries by releasing hatchery produced juvenile fish or invertebrates have been made for more than 150 years [3–6]. However, naïve, inexperienced individuals raised under laboratory or controlled conditions often demonstrate aberrant behaviours, such as reduced antipredator behaviour and foraging in the wild [7–9]. Release experiments have shown that hatchery produced juveniles are more prone to predation when released in the wild [10–12], thus the main challenge is to increase survival rates after release.

The European lobster (*Homarus gammarus*) fishery in Norway has historically been important, but the stock collapsed in the 1960s and has since failed to recover [13]. This gave incentive to supplement the natural stock by releasing hatchery-reared juveniles, a method applied in other species to counter the effects of over-fishing and recruitment failure [3, 14, 15]. Hatchery-produced lobsters have been occasionally released along the Norwegian coast over the past 100 years with variable success [9, 16–18]. Although released lobsters have proven to survive, reproduce and contribute to enhance wild populations, the newly released lobster juveniles seem to have less developed antipredator and shelter seeking behaviour [19]. This has been suggested to be caused by the lack of environmental complexity in the hatchery [12, 19]. In lobster hatcheries, the larvae are kept in incubators for the first three moults. Cannibalism is a problem in intensive culture, thus metamorphosed juveniles (stage IV) are kept in single compartments [20]. The compartments prevent physical interactions with conspecifics and do not contain any substrate or shelter. Shelter-seeking behaviour is presumably a critical factor for survival of lobsters in the wild, thus facilitating the development of such behaviour should be given focus when producing juveniles for releases.

In the present study, juveniles were exposed to two different environments. In the naïve treatment, the lobsters were kept in single compartments under ordinary hatchery conditions. In the exposed treatment, juveniles experienced structural complexity as well as interactions with conspecifics to simulate a natural environment. We made detailed behavioural analyses of both single lobsters and pair-wise interactions between naïve and exposed lobsters. Finally, mixed groups were introduced to a semi-natural environment with competition for shelters. Exposure to structural complexity and social interactions was predicted to make lobsters find and establish themselves in a shelter more rapidly. We further predicted that experience with conspecifics would make exposed lobsters superior in contests over shelters.

## Materials and Methods

Juvenile European lobster of six months of age were purchased from Norwegian Lobster Farm AS (NLF) at Kvitsøy, Norway (59°24´09N 05°24´09E). The broodstock consisted of about 100 females. The juveniles had been raised in single compartments at a temperature of 19 to 21°C and transported to the Institute of Marine Research (IMR) field station at Parisvatnet, Øygarden, Norway (60°37′45″N 04°48′07″E). Mortality and claw loss during transport was low (1.2 and 2.4%, respectively). The juveniles were held outdoors in six flow-through holding tanks (4000 L fiberglass) during a nine days acclimation period before exposure to the treatments. The tanks were provided with sea water from three meters depth (from Nautnesvågen 60°37′42″N 04°47′36″E), filtrated through a macro grid and a 20 μm drum filter. The lobsters were held at a photoperiod of 20L:4D (simulating summer conditions). During the treatments and experiments from 25^th^ of May to 14^th^ of August 2011 ambient water temperature ranged from 8.5 to 18.0°C (mean 13°C) and salinity ranged between 34.5 and 35.0. All lobsters were fed twice a week with frozen krill (Euphasiidae sp.) or formulated feed patented by NLF and produced by the Norwegian Institute of Food, Fisheries and Aquaculture Research (Nofima). Carapace length (CL) was recorded to closest mm below with a vernier caliper from the base of the eye socket to the posterior-medial edge of the cephalothorax. A total of 240 juveniles, average CL of 14.6 mm ± 1.2 (± SD), were then equally divided between the two treatments.

### Treatment conditions

**Fig 1:**
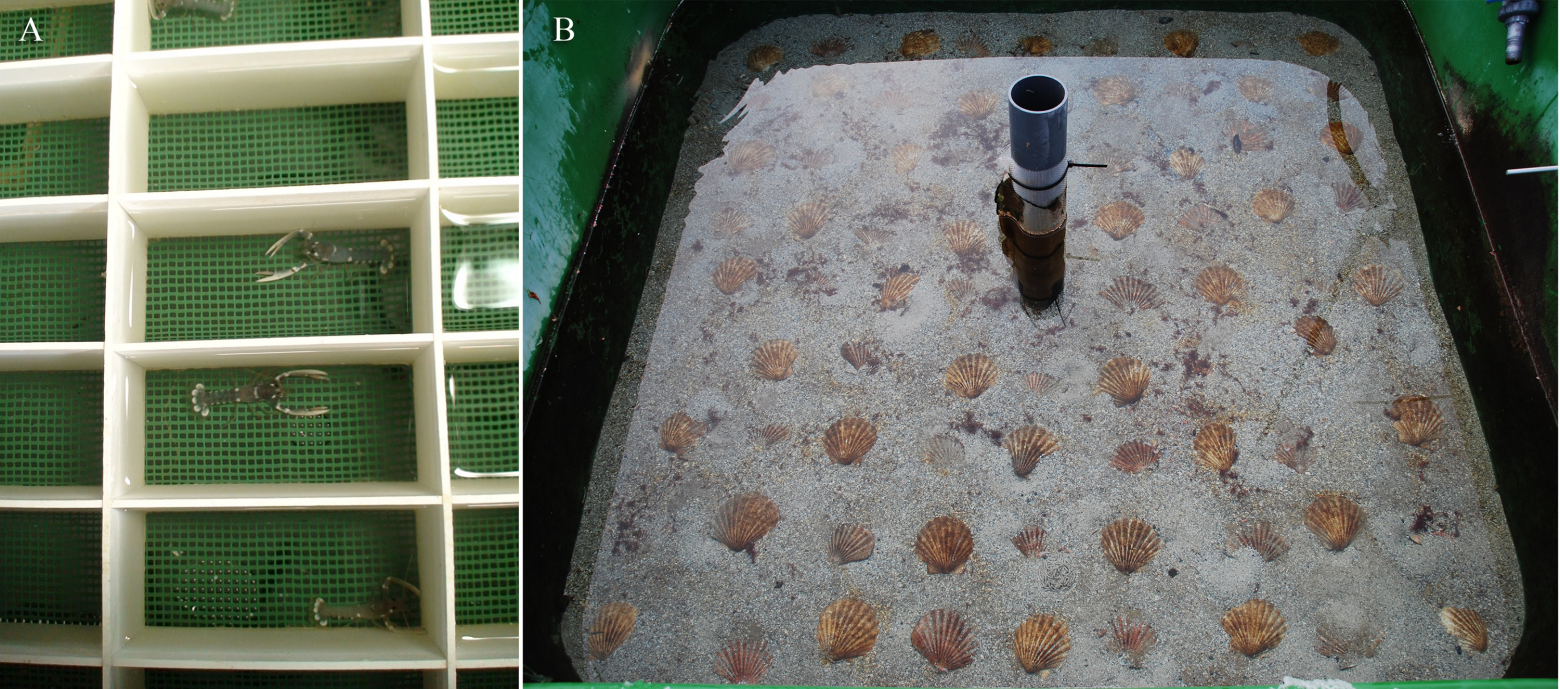
The two different treatments given the European lobster juveniles (*Homarus gammarus*). (A) naïve treatment; trays with single compartments and (B) exposed treatment; shell sand substrate, contact with conspecifics and excess of shelters.

In the “naïve” treatment a total of 120 juveniles were kept in single compartments (36 cm^2^) arranged in trays floating in three of the holding tanks with 40 lobsters in each tank. The treatment period lasted 45 days (25^th^ May to 9^th^ July). Each compartment consisted of solid plastic walls with no cover and a 1 mm^2^ mesh bottom to ensure water exchange. The juveniles were deprived of other stimuli such as substrate, shelter and physical contact with conspecifics (Fig 1A). The naïve juveniles suffered no mortality during the treatment.

In the “exposed” treatment during the same period, 120 juveniles were introduced to three of the holding tanks with 40 lobsters in each tank. The experimental tanks were 2x2 m with rounded corners, which gives an area a bit less than 4m^2^. The release density (10–12 m^-2^) was in accordance with previous studies ([21]; Agnalt, personal communication). The bottom of the tanks was covered with 6–7 cm of coarse shell sand to promote digging. Shelters were provided in excess with 50 valves of *Pecten maximus* in each tank (Fig 1B). The juveniles were released simultaneously at the surface with equal opportunities to find shelter. Of the 120 juveniles, 17 lobsters died during the 45 days of treatment.

### Behaviour experiments

The behaviour studies were conducted indoors in two observation units (50 x 40 x 25 cm; length x width x height). The bottom was covered with coarse shell sand with a single shelter in one end made of three cobble stones (about 5 cm diameter each) and one flat stone (about 17 x 5 cm). The shelters in the behaviour studies differed from the exposure environment in order to provide neutral ground for juveniles from both treatments. Cobblestones are a preferred habitat both for juvenile and adult European lobster ([21]; Agnalt unpublished). A camera (480 TVL-PAL) was mounted above each observation unit with the software Geovision GV-1120T used for recordings. The camera was connected to a computer located in an adjacent room to reduce disturbance. All observations were made under daylight conditions. Only juveniles with both chelae intact and with hardened exoskeleton were used. Each juvenile was placed in the observation units on the opposite side of the shelter and acclimatized for one minute inside a black cylinder with a fine mesh in one end. The recordings started when the cylinder(s) was gently removed. Between each observation, the water of the units was changed by flow through to remove traces of odour from previous lobsters. After the observation period, the juveniles were put back into their respective treatments, and the exposed juveniles were tagged to prevent multiple testing of an individual. The non-toxic compound Visible Implant Elastomer (VIE) was implanted under the transparent cuticle on the ventral side of the tail with a 0.55mm hypodermic syringe. The time of tagging differed slightly both within and between treatments, but this should not have influenced the results, as elastomer tagging does not affect survival, growth or behaviour in European lobster [22–24].

### Single juvenile experiment

One naïve or one exposed juvenile was introduced in each observation unit. The recording time was predetermined to 1200 seconds (20 minutes). To determine if the duration of the treatment had an effect on behaviour, observations were made after 4, 21 and 42 days of treatment. At each observation day, 21 lobsters from each treatment were observed (n = 42).

To quantify the behaviour a number of categories were defined to cover the span of events that could readily be observed in this setting (Table 1).

**Table 1:** Behaviour categories in the single behaviour experiment with European lobster juveniles (*Homarus gammarus*) (adjusted from Huber and Kravitz [25]). Times were recorded in seconds. Total observation time was 1200 seconds.

Behaviour	Description
**Roaming**	Time spent moving around in the observation tank without digging
**Finding shelter**	Time until shelter was approached and touched for the first time without necessarily entering
**Accepting shelter**	Time from release until the lobster was in hiding, i.e. when the posterior tip of the telson and uropods disappeared, or when the lobster turned around to back into shelter with only first pair of chelae and head visible. If the lobster did not accept shelter within 1200 seconds the observation was recorded as not accepted.
**Dwelling**	Time spent inside shelter after acceptance
**Digging**	a. Time spent digging into shelter b. Time spent digging elsewhere than in or around shelter viewed as attempts to acquire an alternative shelter.
**Freezing**	The time the lobster stayed completely motionless on the substrate

### Interaction experiment

After 24 days of treatment one size-matched lobster from each treatment was introduced in an observation unit with a single shelter. A total of 12 pairs of lobsters (n = 24 (12 naïve and 12 exposed)) were observed with each recording predetermined to 1800 seconds (30 minutes). In order to distinguish lobsters from the two treatments in the video recordings each lobster was 24 hours prior to the experiment given a mark on the carapace with a solvent free correction fluid (Pritt Fluid, Solvent-free), one dot for the naïve and two dots for the exposed lobsters.

To determine the winner of the interaction the behaviours of the juveniles were categorized and given scores from -2.0 to +2.0 (Table 2). The scores were summarized for each individual giving a total dominance score, and the lobster with the highest score was defined as dominant. “Fight” represents a high level of aggression and “Evict/Evicted” was also regarded as a strong dominant/subordinate act. “Lunge attack” and “Chase” were given a somewhat lower score. “Threat” performed as an initiation of a fight or as a display to make the opponent retreat was given an even lower score. For “Claw grasp” a physical confrontation of moderate strength and “Fighting in shelter” resulting either in eviction or a successful defence the winner was given +1.0 and the loser -1.0. “Approach” and “Leave shelter” exposing the lobster that left were given a low positive and negative score, respectively. “Slow retreat” was given a score of -1.0. “Rapid retreat”, a stronger response not involving tail-flipping usually displayed by the subordinate as a response to either “Threat” or “Chase”, was given a high negative score. Tail-flip escape, a last resort escape, was given an even higher negative score.

**Table 2:** Behaviour categories in the interaction experiment with European lobster juveniles (*Homarus gammarus*) (modified after Huber and Kravitz [25], Atema and Voigt [26], Cenni et al. [27] and Gherardi et al. [28]). An aggression score is given to each behaviour category. A score of + represents a high level of aggression, 0 is neutral and–represents a low level of aggression.

Behaviour	Description	Score
**Fight**	Pushing, pulling, punching or trying to pinch appendages off the opponent. Often done rapidly and in bursts usually resulting in a tail-flip escape by the opponent	+2.0
**Evict/Evicted**	Walks up to the shelter and forcefully grabs the opponent successfully throwing it out from the shelter (evict). The victim of an eviction gets negative score (evicted)	+/-2.0
**Lunge attack**	Rapid movement towards the opponent	+1.5
**Chase**	Rapid pursuit of a retreating opponent	+1.5
**Threat**	Extends claws and moves them up and down (meral spread)	+1.0
**Claw grasp**	Grasps the opponent’s appendages with the claws or lock one or both claws of the opponent. Positive score to the winner, negative to the loser	+/-1.0
**Fighting in shelter**	Fight that clearly goes on but cannot be described other than by the duration because the animals are completely or partially hidden from view. The winner was the lobster remaining inside the shelter after the fight and got a positive score, the loser got negative score	+/-1.0
**Approach**	Slow advance towards the opponent reducing the distance to less than a body length	+0.5
**Leave shelter**	Leaves shelter without interaction.	-0.5
**Slow retreat**	Moves or turns slowly away from the opponent.	-1.0
**Rapid retreat**	Retreating rapidly from the opponent without tail-flipping	-1.5
**Tail-flip escape**	Contraction of the abdomen to make a rapid escape (swimming/tail-flipping)	-2.0

### Release experiment

The Release experiment was set up to simulate a release scenario, but with no other predators than conspecifics. The experiment lasted 35 days between 10^th^ July and 14^th^ August 2011. Prior to the experiment, the juveniles in the exposure treatment were removed from the tanks for 24 hours. When removing the exposed juveniles it was observed that some were not inside shelter, i.e. “vagrants”. All juveniles were tagged (VIE) prior to the experiment; naïve with orange, exposed with green and vagrants with red VIE. None of the tagged juveniles died or seemed affected by the tagging. The bottom of the experimental tanks was covered with a 6–7 cm layer of shell sand and shelters (scallop valves). In each tank, 27 naïve, 27 exposed and 6 vagrants were released. The juveniles were size matched (Tank 1: naïve CL = 14.8 mm ± 1.2, exposed CL = 15.2 mm ± 1.2, vagrant CL = 15.4 mm ± 0.8; Tank 2: naïve CL = 14.9 mm ± 1.2, exposed CL = 14.9 mm ± 1.3, vagrant CL = 15.1 mm ± 0.8). The number of valves in each tank was 27 resulting in competition for shelter. At days 6, 21 and 35 all lobsters were removed and the shelters displaced so that no shelter would remain in the exact previous position. In addition, all shelter entrances the lobster juveniles had made were destroyed. At each observation time, shelter occupancy and mortality were recorded. After the examination, all juveniles were re-released.

### Statistical analysis

R version 2.15.0 (R Development Core Team 2012) was used for all statistical analyses. Time to accept shelter in the Single juvenile experiment was analyzed using survival analysis with censoring [29]. The variables used in the analysis were the response variable “time to accept shelter”, and the predictors “treatment” and “days of treatment”. Additionally, the survival analysis included a binary variable for the censoring that indicates whether an individual actually accepted shelter within the 1200 seconds of observation or not. Since the variable “days of treatment” only included observations at three different days it was treated as a categorical predictor in the analysis.

Behaviour analyses (Dwelling, Roaming, Digging outside shelter and Digging into shelter) were carried out by using a two-way ANOVA. The behaviour categories were the response variable and “treatment” and “days of treatment” were the predictors. The data for Digging into shelter was analysed with Box-Cox transformed response variable due to differences in variance between the groups.

For the Interaction experiment, the analyses started out testing if individuals that initiated first aggressive display depended on treatment. A generalized linear mixed effect model (GLMM) was applied as the observations were not independent due to grouping of animals into test pairs [30, 31]. Aggression scores and time spent dwelling for the same experiment were analyzed by using a paired Wilcoxon signed rank test and a paired t-test, respectively.

In the Release experiment “treatment” was a fixed effect predictor, and “tank” was a random effect factor. The binary variable “occupying shelter” was the response variable. A GLMM was applied to test the probability of accepting shelter for naïve, exposed and vagrants. The same analysis was used to test for differences in survival at the end of the experiment depending on treatment.

### Ethical note

All experiments were conducted according to regulations for use of laboratory animals (Forsøksdyrutvalget, regulations at www.fdu.no). To reduce stress and mortality, lobster density in the exposure tanks was kept at recommended levels based on previous lobster experiments (Agnalt, unpublished).

## Results

### Single juvenile experiment

After being placed in the observation units, the juveniles generally explored the environment for a few minutes before either entering the cobblestone shelter or digging an alternative shelter. The relative small size of the observation tank probably led to rapid detection of the shelter, and the few lobsters that did not encounter the shelter within the first seconds were largely passive animals that did not explore at all. “Finding shelter” did not yield any differences between treatments (S1 Table) and “Freezing” was seldom displayed thus was omitted from further analysis.

**Fig 2:**
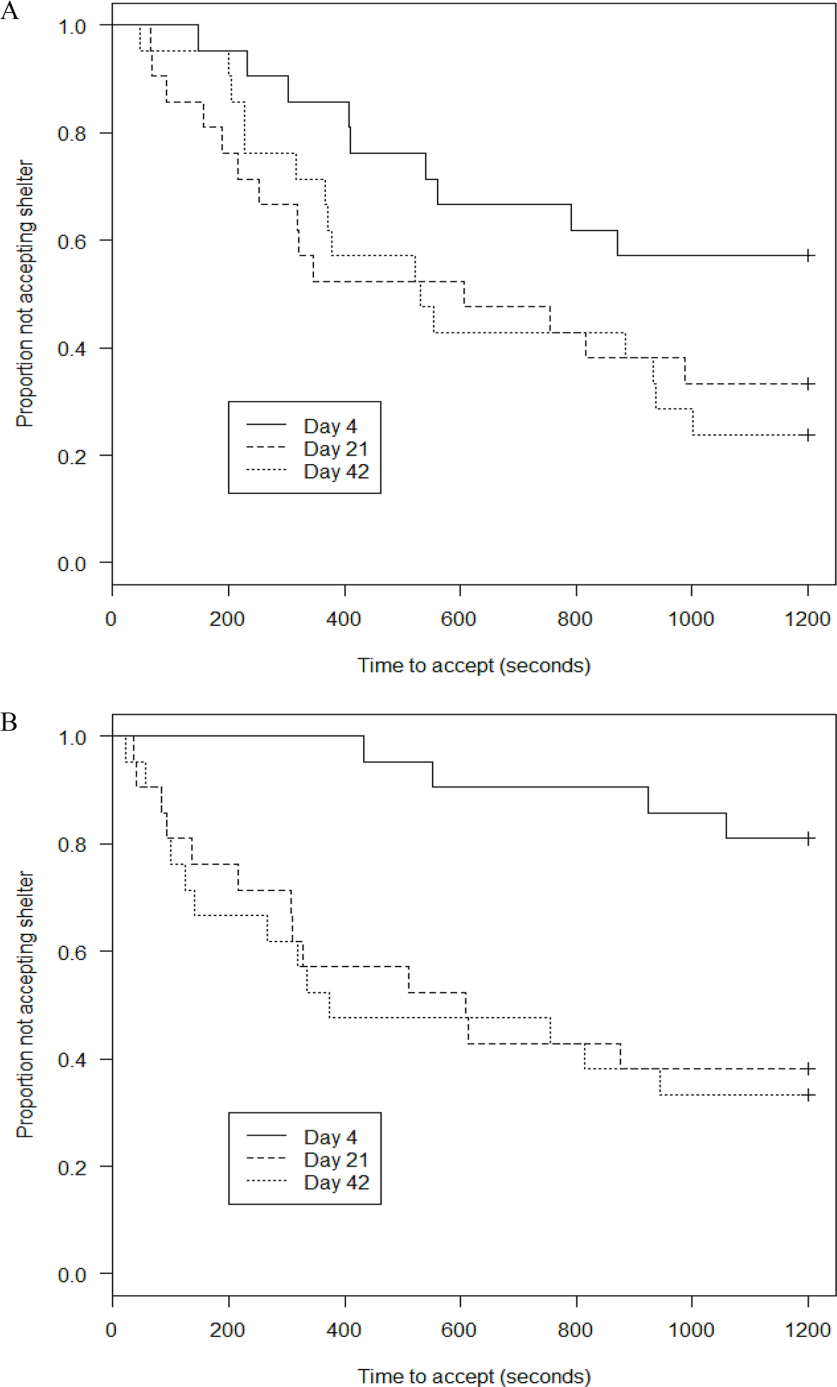
Proportion (%) European lobster juveniles (*Homarus gammarus)* in the single behaviour experiment that did not accept the shelter during the observation time of 1200 seconds, from (A) naïve and (B) exposed treatment.

There were generally few effects of treatment. For several behaviours, there were no differences on any observation day. For “Accepting shelter” there was no interaction between treatment and day of treatment (survival analysis; Chi-square = 2.282, df = 2, p = 0.320) and no overall difference between the two treatments (survival analysis; Chi-square = 0.807, df = 1, p = 0.369, Fig 2A and 2B).

The number of juveniles accepting shelter on days 4, 21 and 42 was similar in the two treatments (naïve: 9, 14, 16; exposed: 4, 13, 14). Median time “Dwelling” in shelter varied from 52 to 73% in naïve juveniles and from 36 to 83% in exposed juveniles with no significant difference between treatments. The number of juveniles “Digging into shelter” on days 4, 21 and 42 was also similar in the two treatments (naïve: 9, 14, 17; exposed: 5, 13, 15). The same was found for median time digging (naïve: 129, 24 and 57 sec; exposed: 83, 21 and 21 sec, F_1,67_ = 2.508, p = 0.118, the data were Box-Cox transformed because of a higher variance in the naïve treatment, Levene’s test; t = 3.233, p = 0.002).

**Fig 3:**
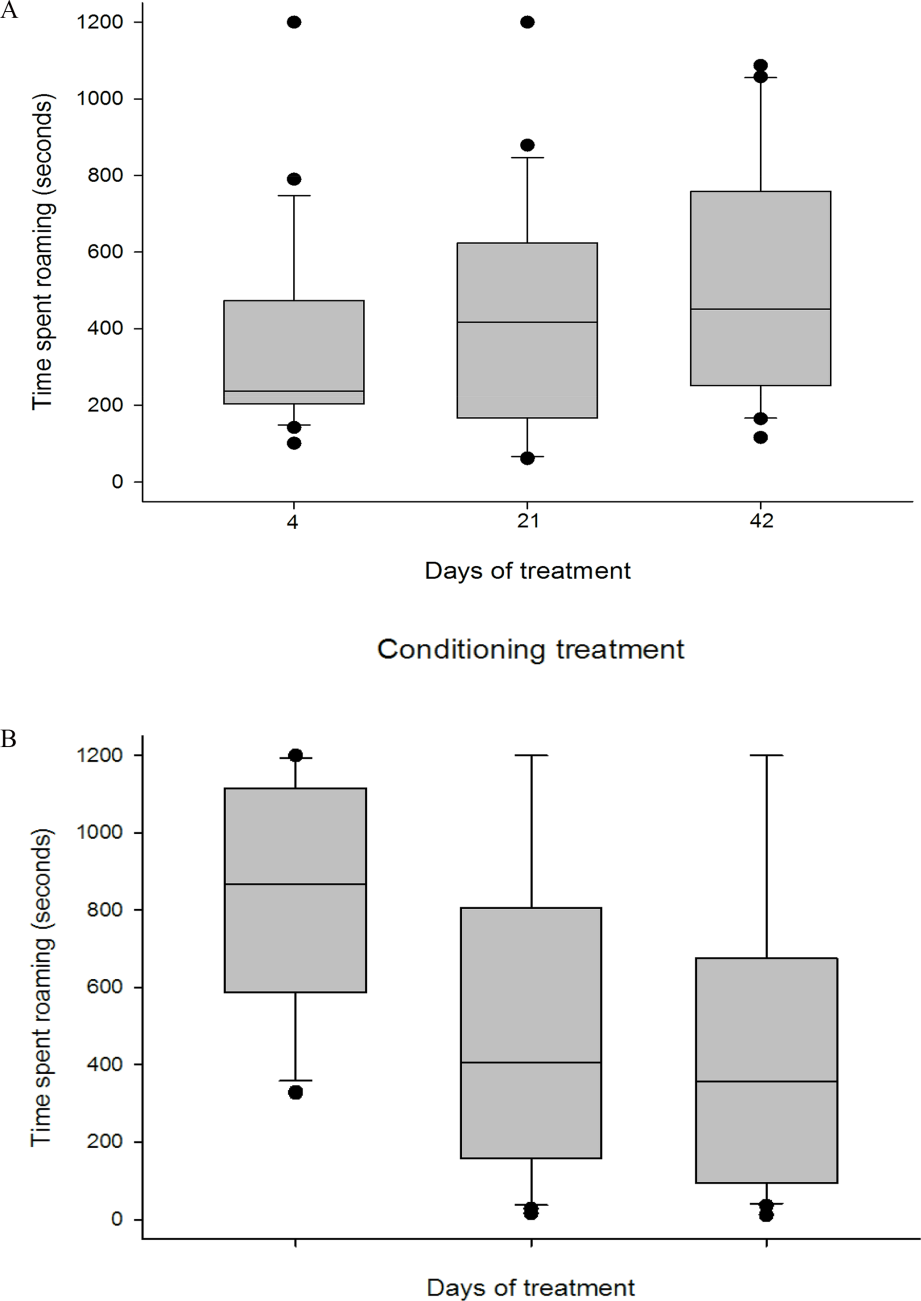
**Time spent “Roaming” (seconds) in European lobster juveniles (*Homarus gammarus*) in the single behaviour experiment tested at treatment day 4, 21 and 42 for (A) naïve and (B) exposed juveniles, n = 21 for each day and treatment.** The data are presented as standard box-and-whisker plot with median, and first and third quartiles. Whiskers represent minimum and maximum values except for outliers.

Some effects of treatment were, however, found. For behaviours outside shelter there were an effect of treatment at day 4 but not at days 21 and 42. The “Roaming” time at day 21 and day 42 varied from 30 to 38% and was similar in the two treatments, but at day 4 exposed juveniles spent significantly longer time “Roaming” than naïve juveniles (72 versus 20% of the total time, t = 4.742, p< 0.001, Fig 3).

“Digging outside shelter” was displayed by 40 of the 126 juveniles (naïve n = 23; exposed n = 17). There was no overall significant difference in mean over the two treatments (F_1,34_ = 3.987, p = 0.054), but at day 4 the naïve juveniles spent almost twice the time digging outside shelter (median 932 sec, 78% of total time) compared to exposed juveniles (482 sec, 40%). On days 21 and 42 the median times were: naïve treatment 652 and 374 sec; exposure treatment 578 and 478 sec.

The duration of the treatments influenced some behaviours. There was a difference in number of juveniles accepting shelter when the two treatments were regarded together between day 4 and 42 (z = 3.696, p< 0.001), while no difference was found between day 21 and 42 (z = 0.418, p = 0.676). The juveniles accepted shelter faster on day 21 than on day 4 (survival analysis; Chi-square = 18.182, df = 2, p< 0.001), and the time “Digging into shelter” decreased from day 4 to day 21 (t = 3.878, p< 0.001). In addition, median time “Dwelling” of exposed juveniles increased from 436 to 893 sec between days 4 and 21 (t = 2.659, p = 0.010) with no further increase on day 42 (995 sec).

### Interaction experiment

**Fig 4:**
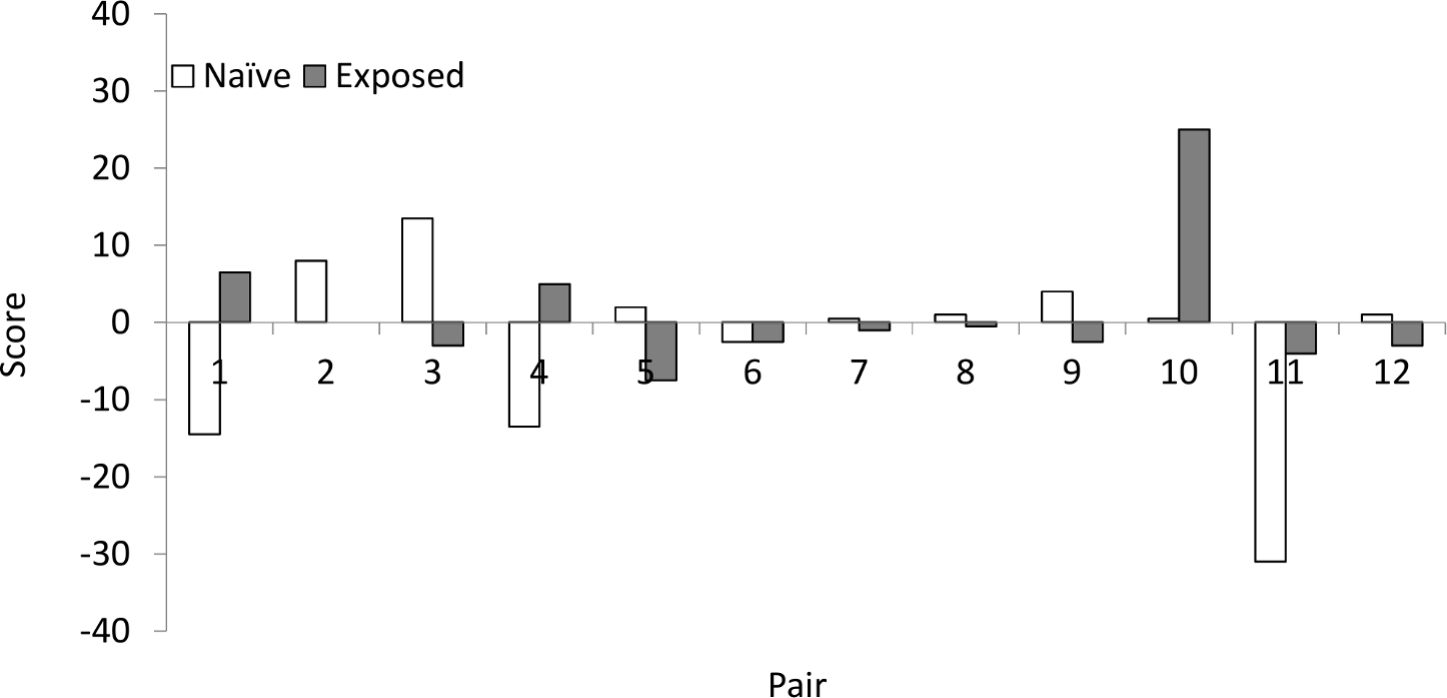
Total behavioural score for each European lobster juvenile (*Homarus gammarus*) from the naïve and exposed treatment in the interaction experiments (n = 12 pairs). The individual with highest score in each interaction was determined as dominant, hence the winner. The experiment was conducted at treatment day 24.

Based on the dominance score more naïve (n = 7) than exposed (n = 4) juveniles were winners (Fig 4; S2 Table), but there was a large individual variation and no significant difference between treatments (paired Wilcoxon signed rank test, V = 38, p = 0.689). In one interaction no winner could be identified. Naïve juveniles initiated, however, significantly more interactions than exposed juveniles did. In the 10 trials with a clear initiator, naïve juveniles initiated eight compared to two in exposed juveniles (GLMM, t = 2.233, df = 11, p = 0.047). Two interactions had no clear initiator.

### Release experiment

**Fig 5:**
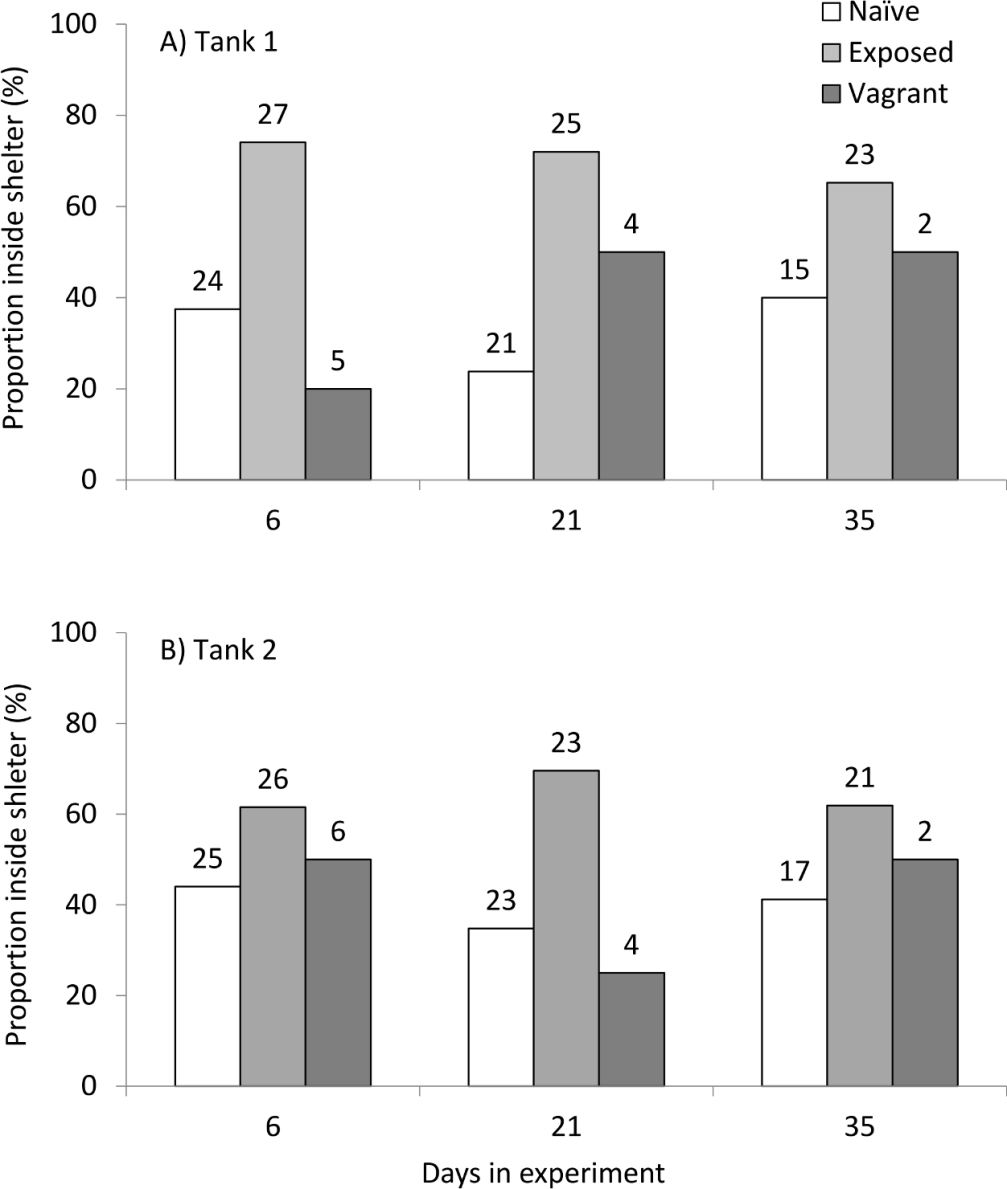
Proportion (%) European lobster juveniles (*Homarus gammarus*) from the naïve, exposed and vagrant treatment found inside shelter at treatment day 6, 25 and 31. Total number of survivors are given above each bar for (A) Tank 1 and (B) Tank 2. 27 juveniles from the naïve and exposed treatment and 6 vagrants were released in each tank at day 1.

**Fig 6:**
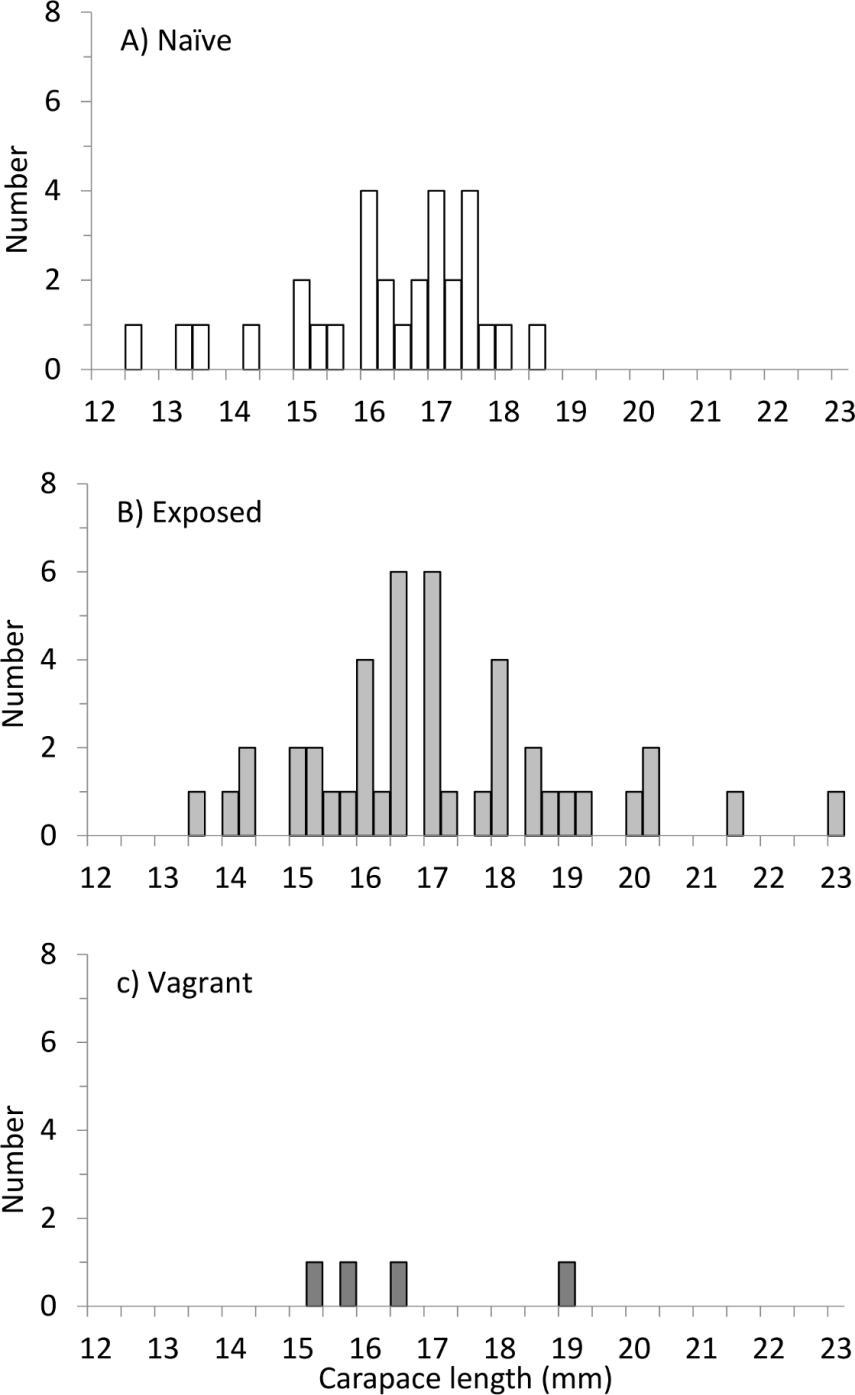
**Carapace length (mm) distribution of the surviving (A) naïve and (B) exposed European lobster juveniles (*Homarus gammarus*) at the end of the release experiment.** The four surviving vagrants are not shown.

Directly following release, there were many interactions both pair-wise and in larger groups, but one hour after release, no interactions were observed. Since the tags were not visible from above the origin of treatment was unknown. At the first observation, day 6, 68% of the exposed lobsters occupied shelters compared to 41% of the naïve juveniles, and the same pattern was found at day 21 and 35 (Fig 5). Exposed juveniles occupied overall statistically significantly more shelters than naïve ones (GLMM, t = 2.667, df = 286, p = 0.008). The exposed juveniles also occupied more shelters than the vagrants but the difference was not statistically significant (GLMM, t = 1.815, df = 286, p = 0.071). When comparing vagrants vs naïve, there was no difference (GLMM, t = 0.264, df = 286, p = 0.792). The survival of naïve juveniles was significantly lower than that of exposed juveniles at the end of the experiment (59% versus 82%, GLMM, t = 2.447, p = 0.016). The vagrants had even lower survival (33%), where the survival was statistically lower than the exposed group (GLMM, t = 3.044, df = 116, p = 0.003) but not the naïve group (GLMM, t = 1.569, df = 116, p = 0.119). The exposed juveniles were also larger at the end of the experiment compared with the naïve juveniles (mean CL = 17.2 mm CL±2.0, mean CL = 16.4±1.6 respectively, Fig 6; S3 Table).

## Discussion

In the Single juvenile experiment, there was no positive effect on shelter seeking behaviour by experience with habitat complexity. In the Interaction experiment, no significant difference in dominance between the two treatments was found. In contrast to this, the exposed juveniles in the Release simulation occupied significantly more shelters than naïve juveniles at all three observation times demonstrating increased competitive and shelter-seeking ability. The survival was also higher in exposed juveniles.

### Experimental set-up

One problem with our design is that we do not know which kind of enrichment is more relevant, as we mixed them when increasing the complexity. On a practical side one may want to do the minimum, and if only putting a shelter or only create the competitive context would be sufficient for improving the release success; this would be less costly and time consuming. However, in the exposed treatment experience with other lobsters were regarded as crucial as lobsters do interact upon release in the wild [12]. Shelters and substrate were provided as lobsters are cannibalistic and survival without hiding places was expected to be low [32–34]. Lobsters are regarded as ferocious cannibals [20], but the low mortality of 14.2% during the exposure treatment demonstrates that juvenile lobsters can coexist in a limited area as long as densities and size differences are moderate and substrate, shelter and feed are provided in excess.

Size, molt stage and memory of prior experience influence the outcome of agonistic encounters in lobsters [26, 35, 36]. The first two factors can be ruled out here as carapace length was matched in all experiments and only hard shelled animals were used. The naïve and exposed juveniles had never encountered each other before, thus the outcome could not be related to memory of prior experience.

### Single juvenile and Interaction experiments

Habitat complexity did not trigger any significant behaviour differences in the Single juvenile experiment, except for a difference after four days of treatment. Exposed juveniles then spent more time “Roaming” compared with naïve juveniles and accepted shelter less often (although not significant). This difference could be due to stress caused by moving the exposed juveniles to a new environment only four days ahead of the first test while the naïve juveniles had been kept in the same environment since stage IV larvae. Some changes over time could not be explained by an effect of treatment. When the time of treatment increased from 4 to 21 days the juveniles in both treatments accepted shelter faster, used less time to dig into shelter and exposed juveniles also spent more time in shelter. One reason for this could be that the temperature increased from 9 to 12.5°C from day 4 to day 21.

Hence, no clear positive effect on shelter-seeking behaviour by experience with habitat complexity was found. In behaviour studies of adult American lobster the exploration time when introduced individually into a new environment did not differ between communally reared lobsters and lobsters reared in solitary confinement [37]. As naïve juveniles displayed similar digging and shelter exploration as exposed juveniles, this behaviour seems to have strong innate components [38]. In contrast, Carere et al. [39] found that European lobsters reared with shelter had higher sheltering scores than those reared in non-enriched conditions. The reason for difference is not completely clear, but in contrast to Carere et al. who exposed lobsters to similar shelters during the enrichment and the subsequent tests, the shelters in our behaviour studies differed from those in the exposure environment. This may be more relevant for an actual release in the wild where lobsters encounter various types of shelters.

In the Interaction experiment naïve juveniles overall won more interactions, but there was no significant difference in dominance scores between the two treatments. Naïve juveniles initiated significantly more first aggressive encounters. Without previous experience with interactions, they may have initiated encounters as a defence mechanism. Previous studies have also found that juvenile naïve lobsters readily engage in agonistic encounters [25]. Aggressive behaviour could also make the naïve juveniles more vulnerable to predators and may partly explain the great loss when releasing hatchery reared lobster juveniles to the wild [12].

### Release experiment

In contrast to the lack of significant effects of treatment in the previous behaviour experiments, the exposed juveniles in the Release experiment occupied significantly more shelters than the naïve juveniles at all three observation times. The higher overall survival of exposed than naïve juveniles strongly suggests that shelter is a crucial resource.

Many agonistic encounters involving two or several lobsters were observed shortly after release but not later, indicating that dominance was settled within the first hours and maintained in subsequent encounters by displays rather than physical fights (see also [26]). The initial occupant has a resident advantage over an intruder in terms of shelter dominance [40], and some occupant advantage was likely present in the last two observations of the present study when territories and hierarchies were already established. The shelters were slightly displaced after each observation and all entrances to the shelters destroyed, but the three observation days were still not strictly independent. However, a difference between treatments was observed already at the first observation time.

## Concluding remarks

An enriched environment did not improve shelter-seeking behaviour in lobster juveniles when tested alone in a small tank, but when a large number of lobsters competed for a limited number of shelters in a semi-natural environment, experience of environmental complexity and interactions with conspecifics had a clear positive effect. Studies under more realistic conditions are thus needed to demonstrate the effect of different treatments.

To reduce the running costs of producing exposed lobster juveniles for stock enhancement or sea ranching future studies should focus on optimizing lobster density and the duration of the exposed period. Densities higher than 10–12 lobsters m^2^ as used here could make the exposure period more cost-efficient, and survival could be further increased if a minimum exposure time is established. The stress of newly being exposed to the exposed environment (interactions with conspecifics) may explain why exposed juveniles spent longer time “Roaming” outside shelter than naïve juveniles at day 4 while at day 21 the juveniles had adapted to their new environment and at this point no difference was found. This indicates that the minimum exposure time is between 4 and 21 days, which is shorter than the earlier studied exposure time of 47 days (Agnalt, personal communication).

As extrapolations from laboratory results to the wild should be done with caution [1, 41], a small-scale study observing the behaviour of exposed lobsters after release is needed to confirm our findings. If further experiments show that experience with substrate, shelter and conspecifics improves lobster juvenile shelter-seeking behaviour and survival, exposure before release should have the potential to improve the outcome of stock enhancement and sea ranching.

## Supporting Information

S1 Table Single behaviour experiment with European lobster *Homarus gammarus*. Listed are the different categories used to compare lobster juvenile behaviour in the two treatments “naïve” and “exposed”. In the “naïve” treatment, the juveniles were raised in single compartments without substrate and shelter whereas juveniles in the “exposed” treatment experienced substrate, shelter and interactions with conspecifics. Individual lobster juveniles (ID) were tested at day 4, 21 and 42. Carapace length (CL) was measured at each test day. Time was recorded in seconds and total observation time was 1200 seconds.
(XLSX)

S2 Table Interaction experiment with European lobster juveniles *Homarus gammarus*. Behaviour categories with aggression score given for the 12 pair of lobster juveniles tested. Each pair was size matched (carapace length, CL) and consist of one “naïve” and one “exposed” lobster juvenile that was observed for 1800 seconds. In the “naïve” treatment, the juveniles were raised in single compartments without substrate and shelter whereas juveniles in the “exposed” treatment experienced substrate, shelter and interactions with conspecifics. A score of + represents a high level of aggression, 0 is neutral and–represents a low level of aggression.
(XLSX)

S3 Table Release experiment with European lobster *Homarus gammarus*. Carapace length (CL) in mm of the lobster juveniles at the start and at the end of the release experiment. Two experimental tanks, UB-1 and UB-2, were used. In the “naïve” treatment, the juveniles were raised in single compartments without substrate and shelter whereas juveniles in the “exposed” treatment experienced substrate, shelter and interactions with conspecifics. “Vagrants” were juveniles that was found outside shelter in the “exposed” treatment. The lobster juveniles were tagged with three different coloured VIE tags before released into the experimental tanks.
(XLSX)
